# Integrated Bile Metabolomics and Serum Biomarkers for Differentiating Cholangiocarcinoma from Cholelithiasis

**DOI:** 10.3390/biomedicines14102356

**Published:** 2026-10-16

**Authors:** Jinduo Zhang, Leiqing Wang, Xu Zhang, Ping Yue, Boru Jin, Ningning Mi, Jinyu Zhao, Long Gao, Dylan Lee, Yanyan Lin, Wenbo Meng

**Affiliations:** 1 Department of General Surgery, The First Hospital of Lanzhou University, No. 1 Donggang West Road, Lanzhou 730030, China; 2 The First School of Clinical Medicine, Lanzhou University, Lanzhou 730030, China; 3 Endoscopic Diagnosis and Treatment Center, Gansu Provincial Hospital, Lanzhou 730000, China; 4 Clinical Research Center, Big Data Center, The Seventh Affiliated Hospital, Sun Yat-sen University, Shenzhen 518107, China; 5 CuraStrategix Health, Aurora, OH 44202, USA; 6 Gansu Province Key Laboratory of Biological Therapy and Regenerative Medicine Transformation, Lanzhou 730030, China

**Keywords:** cholangiocarcinoma, cholelithiasis, bile metabolomics, diagnostic model, exploratory analysis, pyruvate

## Abstract

**Background:** We investigated bile metabolites and serum variables for differentiating cholangiocarcinoma (CCA) from cholelithiasis. **Methods:** Bile from 31 patients with CCA and 48 with cholelithiasis was profiled by high-resolution LC-MS. Candidate metabolites and serum variables were integrated in a seven-variable classifier, and public single-cell data provided tissue-level biological context. **Results:** Of 2269 annotated metabolite features, 532 met the exploratory screening criteria. The selected signature comprised pyruvic acid, lipoxin A4, CEA, APOA1, 3-chloro-L-tyrosine, ADP-ribose, and glutaric acid; pyruvic acid had the largest contribution in the SHAP analysis. Random forest achieved an AUC of 0.894 in the classifier comparison, a training cross-validation AUC of 0.820, and an AUC of 0.914 in the 16-patient test subset. Because selection used all 79 patients before evaluation, these internal performance estimates may be optimistic. **Conclusions:** Integrated bile and serum profiling identified a promising seven-variable signature for differentiating CCA from cholelithiasis in this cohort. Added diagnostic value over routine serum markers and performance in other benign biliary conditions have not been established.

## 1. Introduction

Cholangiocarcinoma remains one of the most diagnostically challenging hepatobiliary malignancies [1,2,3]. At the time treatment decisions are made, it typically presents within a field of overlapping findings—biliary obstruction, cholangitis, stone-related disease, inflammatory strictures, and nonspecific hepatic lesions—and early disease often lacks pathognomonic imaging [4,5,6,7,8,9,10]. Serum CA19-9 and related markers are clinically useful but confounded by cholestasis, infection, and inflammation [11], whereas endoscopic brush cytology and intraductal biopsy are limited by sampling error, low cellular yield, and desmoplastic architecture [12]. Diagnosis is therefore usually reached by convergence of imperfect evidence rather than by a single decisive test, creating a need for complementary molecular information that reflects the local lesion.

Compared with peripheral blood, bile is the direct microenvironment of the biliary epithelium and is less diluted by systemic background, making it a logical lesion-proximal source of diagnostic metabolites [13,14]. Bile composition shifts under malignant obstruction, cholestasis, epithelial injury, inflammation, and tumor-associated metabolic reprogramming [15,16,17,18,19,20]. No single metabolite is likely to suffice, and discovery-phase signatures often lose performance when tested in independent cohorts [21,22,23,24,25,26]; a clinically useful signature must therefore be compact, reproducible, and interpretable, rather than a long inventory of differential features.

We profiled bile from 31 patients with CCA and 48 with cholelithiasis and explored a model integrating bile metabolites with routine serum variables. Public single-cell RNA-sequencing data were examined separately for tissue-level context [27,28]. This discovery comparison does not test the indeterminate-stricture population in which an adjunctive diagnostic test would ultimately be used.

## 2. Methods

### 2.1. Ethics Approval, Study Population, and Bile Collection

This retrospective observational study was approved by the Human Research Ethics Committee of the First Hospital of Lanzhou University (LDYYLL2026-538) and conducted in accordance with the Declaration of Helsinki. The requirement for informed consent was waived because the study was based on de-identified clinical data and residual specimens collected during routine medical care.

We included 31 patients classified as CCA and 48 patients diagnosed with cholelithiasis. Among the CCA cases, 29 had adenocarcinoma, one had adenosquamous carcinoma, and one was classified on clinical and imaging grounds without histologic confirmation because that patient did not undergo surgery (Supplementary Table S3). Twenty-three patients underwent resection and had pathologic stage information; eight did not undergo resection. The CCA locations were two intrahepatic, ten perihilar, and nineteen distal. Only two intrahepatic cases were available, precluding a meaningful location-stratified metabolomic comparison. The benign comparator comprised patients with stones, some of whom had cholangitis; no PSC, IgG4-related cholangitis, or indeterminate-stricture controls were included.

Bile (1–6 mL) was collected via endoscopic nasobiliary drainage or intraoperatively, transported on ice, centrifuged at 3000× *g* for 15 min at 4 °C, aliquoted, and stored at −80 °C. Bile was obtained endoscopically from 8 CCA and 20 control patients, and intraoperatively from 23 CCA and 28 control patients (28 endoscopic and 51 intraoperative samples in total). Prior biliary stenting or drainage was recorded in 10 CCA and 4 control patients. Acute cholangitis at sampling was noted in 3 CCA and 18 control patients. At admission, before biliary decompression, 4 CCA and 3 control patients had peak total bilirubin ≥34.2 µmol/L. Table 1 reports serum bilirubin measured on the day of bile collection after decompression, rather than these admission peak values: mean ± SD was 14.2 ± 2.2 µmol/L in CCA and 12.1 ± 3.5 µmol/L in controls. Sampling-day serum values, including bilirubin, CEA, APOA1, GGT, and CA19-9, were used for modeling.

### 2.2. UHPLC-MS/MS-Based Metabolomic Profiling

Bile samples were thawed on ice before analysis. For metabolite extraction, 50 μL of each sample was mixed with 150 μL acetonitrile:methanol (1:4, *v*/*v*) containing internal standards, vortexed thoroughly, and centrifuged at 12,000 rpm for 10 min at 4 °C. The supernatant was then incubated at −20 °C for 30 min to enhance protein precipitation, followed by a second centrifugation at 12,000 rpm for 3 min at 4 °C. A 120 μL aliquot of the clarified extract was transferred for LC-MS analysis.

Metabolomic profiling was performed using a Nexera UHPLC system (Shimadzu, Japan) coupled to an Orbitrap Exploris 480 mass spectrometer. Chromatographic separation was achieved on an ACQUITY UPLC BEH C18 column (150.0 mm × 2.1 mm, 1.7 μm) maintained at 40 °C. The mobile phases consisted of water containing 0.2% acetic acid (phase A) and methanol containing 0.2% acetic acid (phase B), delivered at a flow rate of 0.35 mL/min. The gradient program was as follows: 10% B from 0 to 1.0 min, 10–95% B from 1.1 to 8.0 min, 95% B from 8.1 to 9.0 min, 95–10% B from 9.1 to 9.2 min, and 10% B from 9.3 to 12.0 min. MS1 and MS2 spectra were acquired in both positive- and negative-ion modes.

Given the analytical platform, high-resolution MS acquisition, and subsequent database-supported metabolite annotation strategy, the present workflow was treated as high-resolution LC-MS metabolomic profiling with broad metabolome coverage rather than as a quantitative targeted assay. The term “wide-targeted,” where used, refers only to the operational breadth of metabolite coverage in the analytical workflow and should not be interpreted as predefined MRM-transition-based quantification.

### 2.3. Metabolomic Data Processing, Metabolite Annotation, and Pathway Analysis

Raw LC-MS data were subjected to peak extraction, alignment, noise filtering, and retention-time correction. Metabolite peak areas were normalized to the corresponding internal standard peak area to reduce variation related to extraction recovery and instrument response. Features with a signal-to-noise ratio >3 were retained for downstream analysis.

The metabolite matrix had less than 5% overall missingness. Missing values were imputed by k-nearest neighbors (k = 5), followed by log transformation and unit-variance scaling.

Unsupervised principal component analysis (PCA) and hierarchical clustering were used to characterize the global structure of the metabolomic data. Orthogonal partial least-squares discriminant analysis (OPLS-DA) was subsequently performed as a supervised multivariate approach, and model robustness was assessed by 200-permutation testing. In the present study, OPLS-DA was used as an exploratory discrimination method rather than as a stand-alone inferential framework. Differential metabolites between groups were identified using a combined threshold of variable importance in projection (VIP) > 1 and *p* < 0.05 by Student’s *t* test. VIP values were derived from the OPLS-DA model, and the corresponding score and permutation plots were generated in MetaboAnalystR. Because this screening strategy was used for exploratory differential analysis and downstream feature prioritization, the resulting metabolite set was interpreted in conjunction with multivariate structure, pathway context, and model-based feature selection rather than as a definitive list of independently validated biomarkers.

Metabolite annotation was performed by accurate mass matching with a mass error threshold < 5 ppm, together with MS/MS spectral matching against the HMDB and KEGG databases. Accordingly, metabolite identities were assigned as database-supported putative annotations rather than absolute structural confirmations. Pathway enrichment analysis was conducted using MetaboAnalyst 5.0, with *p* < 0.05 considered statistically significant. The KEGG library used in MetaboAnalyst 5.0 corresponded to the 9 August 2020 update, and the HMDB library corresponded to the 9 August 2021 update/HMDB 5.0. Pearson correlation analysis was used to evaluate associations among selected metabolites.

### 2.4. Quality Control of Metabolomic Profiling

QC samples were prepared by pooling equal aliquots of 10 μL from the extract of each biological sample after sample pretreatment. The pooled QC sample was analyzed together with the study samples and used to monitor instrument stability, retention-time reproducibility, and analytical repeatability.

Data from each analytical mode were processed and quality-controlled independently, and metabolite features were compared between study groups only within the same analytical mode. The different analytical profiles shown in the QC chromatograms therefore reflect the use of complementary acquisition conditions to broaden metabolome coverage, rather than the mixing of raw feature data across incompatible chromatographic settings.

Analytical quality was assessed using several complementary quality-control measures, including total ion chromatogram overlap, peak-area reproducibility, internal-standard stability, and inspection of blank samples for potential interference. The prespecified quality-control criteria were total ion chromatogram overlap >90%, peak area relative standard deviation <15%, internal-standard relative standard deviation <10%, and absence of notable interfering peaks in blank samples. These quality-control procedures were used to verify the overall analytical stability of the metabolomic platform before comparative and modeling analyses were performed.

### 2.5. Public scRNA-Seq Datasets

Public single-cell RNA-sequencing datasets were retrieved from the Gene Expression Omnibus (GEO), with selection restricted to human CCA tumor tissues and paired adjacent tissues. Dataset inclusion prioritized studies that enabled interrogation of tumor microenvironment heterogeneity and metabolism-related transcriptional programs. Three datasets were included: GSE213452, GSE181878, and GSE138709.

### 2.6. scRNA-Seq Preprocessing, Integration, and Clustering

Single-cell RNA-sequencing data were processed using Seurat (version 4.0.4). Initial quality control was performed separately for each dataset before integration. Cells were retained if they met the following quality-control thresholds: nCount_RNA between 500 and 20,000, nFeature_RNA between 250 and 6000, and percent.mt ≤ 25%. Genes detected in fewer than three cells were excluded.

Cross-dataset batch correction and integration were performed using the Seurat anchor-based workflow. Briefly, FindIntegrationAnchors was applied using 2000 highly variable genes to identify shared anchors across GSE213452, GSE181878, and GSE138709, followed by IntegrateData to generate an integrated expression matrix with reduced technical batch effects. Principal component analysis was then performed on the integrated object, and the top 50 principal components were used for graph-based clustering at a resolution of 0.6. Uniform manifold approximation and projection (UMAP) was used for low-dimensional visualization.

All downstream analyses, including clustering, cell-type annotation, differential expression analysis, and pathway-level scoring, were performed on the integrated object. These parameters were selected to harmonize the three public datasets while preserving major biological transcriptional structure for downstream interpretation.

### 2.7. Cell-Type Annotation, Differential Expression, and Gene Set Enrichment Analysis

Cluster-defining marker genes were identified using FindMarkers with adjusted *p <* 0.05, and cell identities were assigned on the basis of canonical lineage markers. Major annotated populations included B cells (CD19, MS4A1), cholangiocytes (KRT7, KRT19), endothelial cells (PECAM1, VWF), fibroblasts (COL1A1, DCN), hepatocytes (ALB, APOB), malignant cells (EPCAM, KRT18), myeloid cells (CD14, LYZ), NK cells (NKG7, GNLY), plasma cells (SDC1, IGHG1), and T cells (CD3D, CD4). In the present study, these markers were used for practical annotation of major cellular populations in the integrated atlas; annotation of malignant cells therefore reflects tumor-associated epithelial populations defined by transcriptional context and marker expression, rather than formal copy-number-based malignancy inference.

Cell-level differential-expression screens compared tumor and adjacent tissues within annotated populations, focusing on epithelial and myeloid cells. We used an absolute log-fold-change threshold of 0.5 and Bonferroni-adjusted cell-level *p* values. Because cells from the same donor are not independent biological replicates, these comparisons are interpreted descriptively. HK1, PKM, and LDHA were examined as candidate glycolysis-related genes.

Exploratory gene-set scoring used MSigDB glycolysis, glucose-metabolism, and pyruvate-metabolism sets and summarized patterns by normalized enrichment score.

### 2.8. Model Development and Statistical Analysis

Candidate bile metabolites and serum laboratory measurements were considered together. Variables with more than 10% missingness were excluded. Remaining metabolite missing values were imputed by k-nearest neighbors and clinical variables by multiple imputation. Group comparisons used Student’s *t* test, the Mann–Whitney U test, or the chi-square test according to variable type and distribution.

Metabolites were screened by VIP and nominal *p* value on the full cohort (*N* = 79), and LASSO selected the candidate variables before the training/test split. Because this outcome-informed selection included patients later used for evaluation, the reported cross-validation and 16-patient test AUCs may be optimistic; they should be interpreted as exploratory internal results. Feature-selection frequency for each retained variable was not available for this revision.

## 3. Results

### 3.1. Study Framework and Clinical Context for Biomarker Discovery

**Figure 1:**
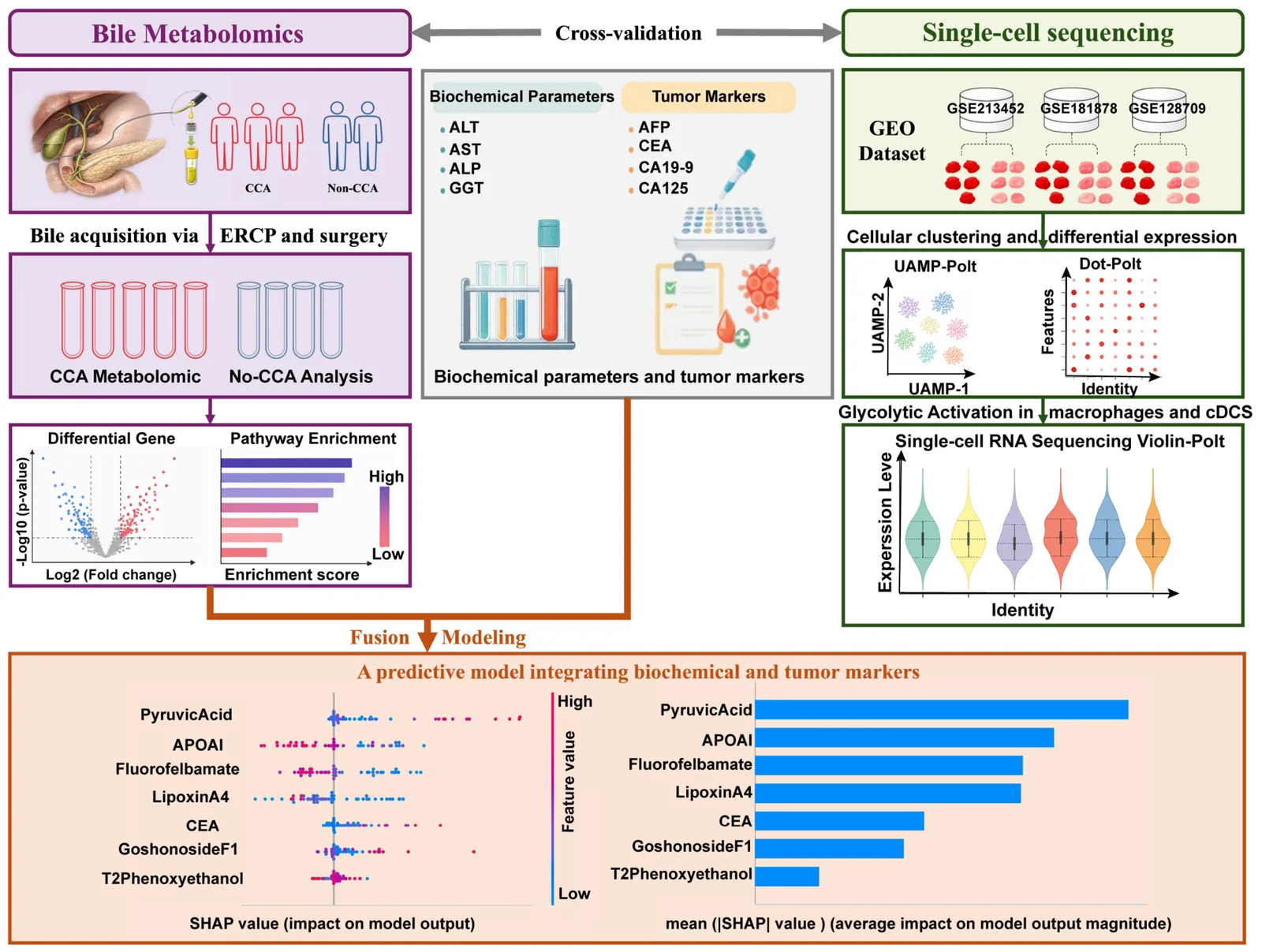
Study workflow. The bile-metabolomic and serum-variable analyses were performed in the 79-patient CCA-versus-cholelithiasis cohort; public tissue single-cell datasets were examined separately. The feature examples drawn within this schematic require updating to match the final selected variables in Section 3.3. In the UAMP and violin plots, the different colors represent distinct cell clusters/types, as indicated on the Identity axis. In the volcano plot, blue dots indicate downregulated features, red dots indicate upregulated features, and gray dots indicate non-significant features.

**Figure 2:**
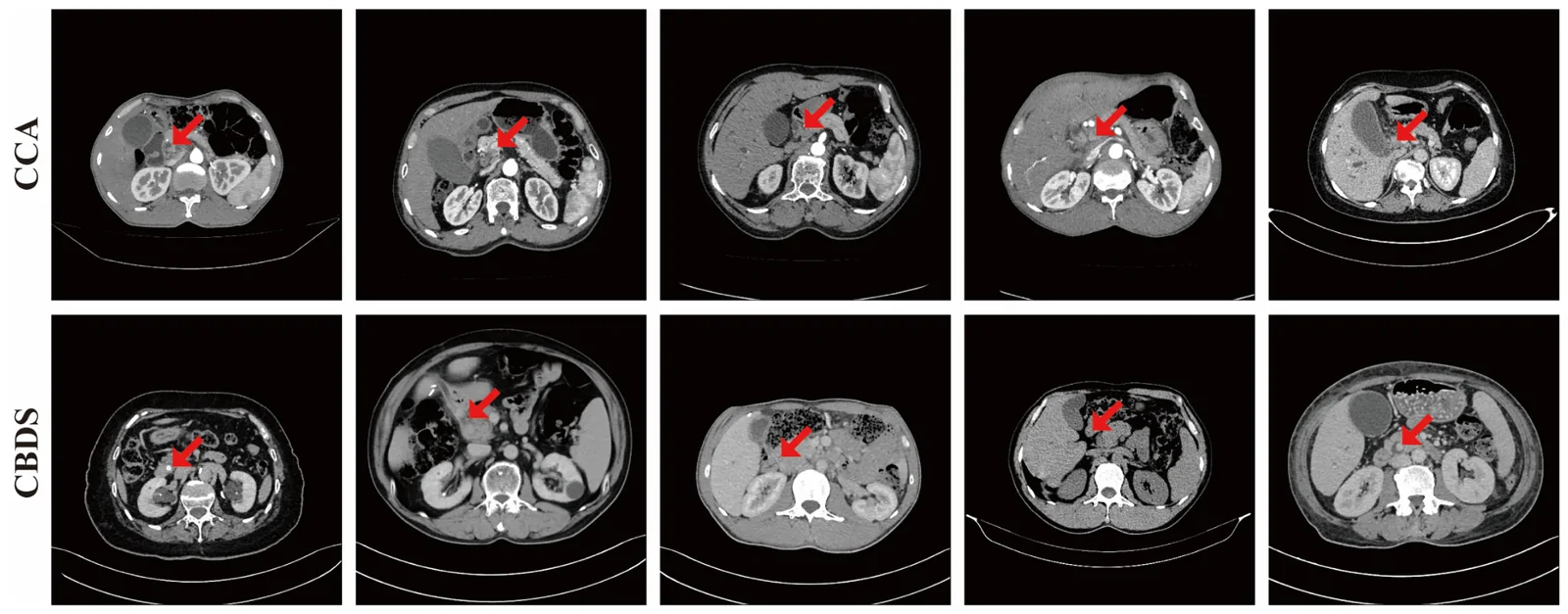
Representative CT images of CCA and common bile-duct stones. Arrows indicate the reported biliary abnormalities. The selected images illustrate appearances in individual patients and do not quantify diagnostic ambiguity.

**Figure 3:**
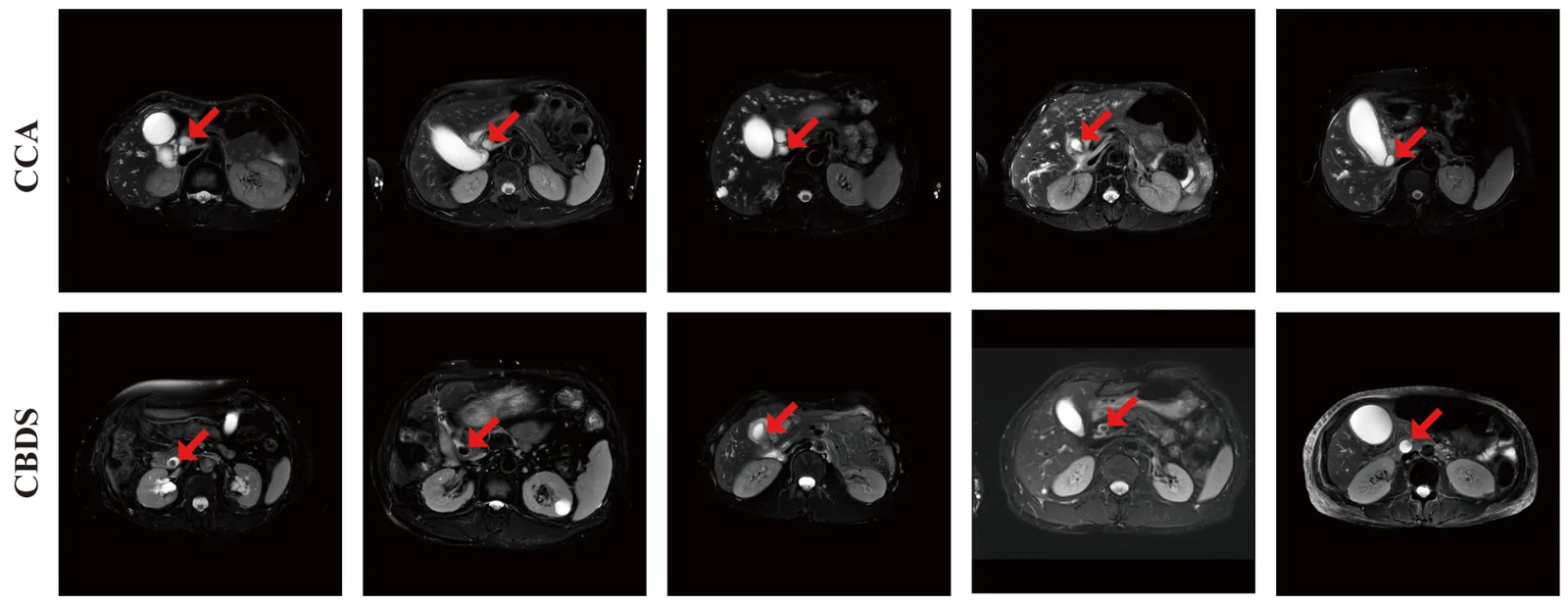
Representative MR images of CCA and common bile-duct stones. Arrows indicate the reported biliary abnormalities. The selected images are illustrative and do not provide a comparative diagnostic-accuracy assessment.

The study combined bile metabolomics, clinical variables, and a separate public single-cell analysis (Figure 1). Representative CT and MR images of CCA and common bile-duct stones are shown in Figure 2 and Figure 3; these illustrations do not establish that the two conditions were diagnostically indistinguishable in the enrolled patients.

### 3.2. Bile Metabolomics Identifies Marked Metabolic Alteration in CCA

**Figure 4:**
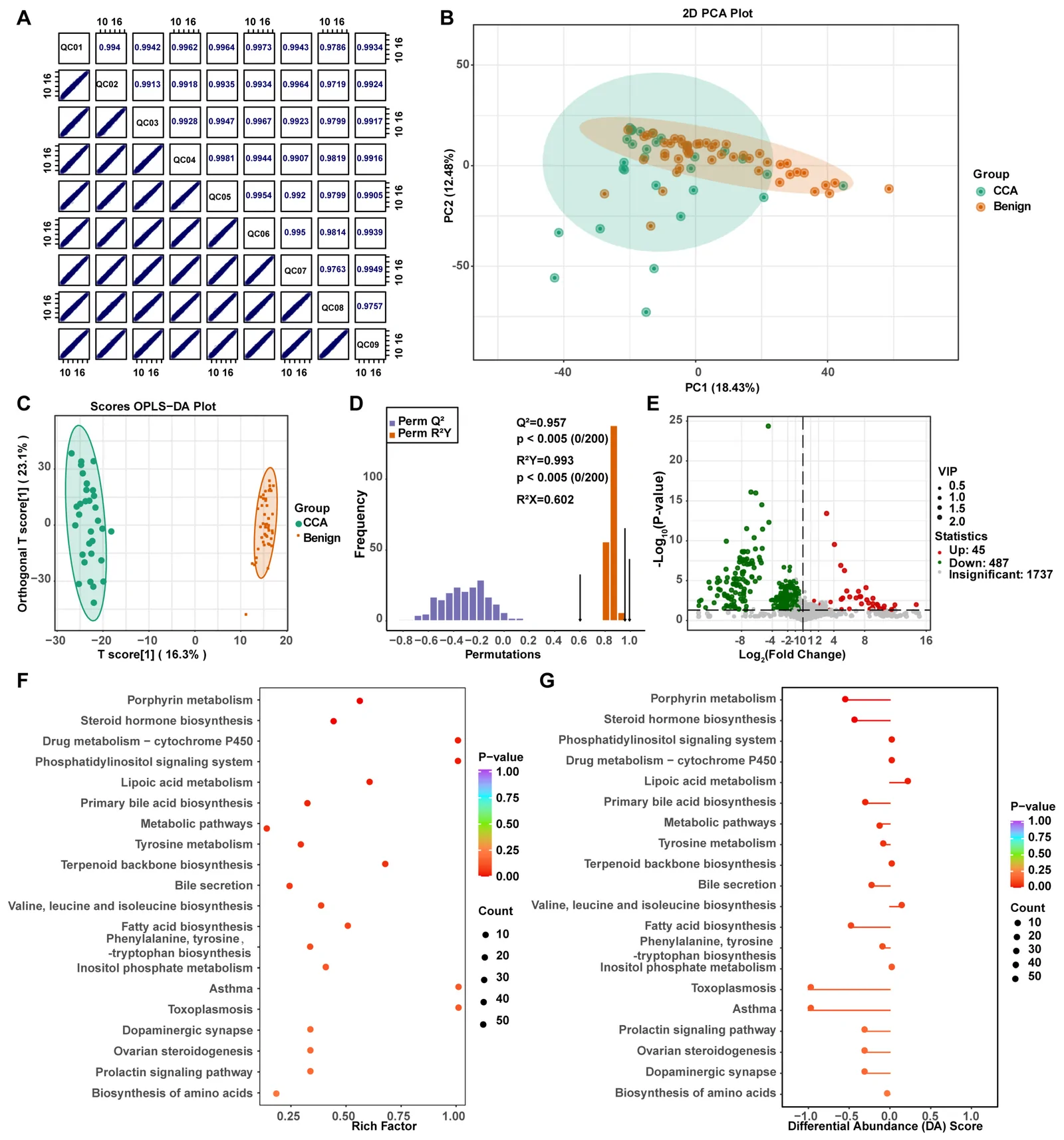
Bile metabolomics in CCA and cholelithiasis. (**A**) Pooled QC correlations; (**B**) PCA; (**C**) OPLS-DA; (**D**) permutation analysis; (**E**) volcano plot; (**F**) KEGG pathway enrichment; and (**G**) differential-abundance scores. In (**D**), arrows indicate the observed Q^2^ and R^2^Y of the actual model; in (**E**), the horizontal dashed line denotes *p* = 0.05, and vertical dashed lines denote the fold-change cutoff.

Across 79 bile samples, high-resolution LC-MS profiling identified 2269 annotated features. Pooled QC samples supported analytical consistency (Supplementary Figure S1; Figure 4A). PCA showed a distributional trend, and supervised OPLS-DA separated the CCA and cholelithiasis groups (Figure 4B–D).

Using VIP > 1 and nominal *p* < 0.05, 532 of 2269 annotated features were selected, including 45 higher- and 487 lower-abundance features in CCA (Figure 4E). The nominal *p*-value threshold was used as an inclusive exploratory screen to prioritize candidate metabolites for subsequent feature selection, rather than as a confirmatory test of each metabolite.

The observed pattern comprised many lower-abundance features and a smaller number of higher-abundance features in CCA. Figure 4F presents pathway enrichment, and Figure 4G summarizes differential-abundance scores. Together, these results identify metabolic patterns associated with the two diagnostic groups.

### 3.3. Integrated Feature Selection Defines a Seven-Variable Diagnostic Signature

We combined bile features with routine biochemical and serum tumor-marker variables, including APOA1, CEA, GGT, AFU, PA, HGB, lymphocyte percentage, CA19-9, and FDP. The selection workflow is described in Section 2.8.

**Figure 5:**
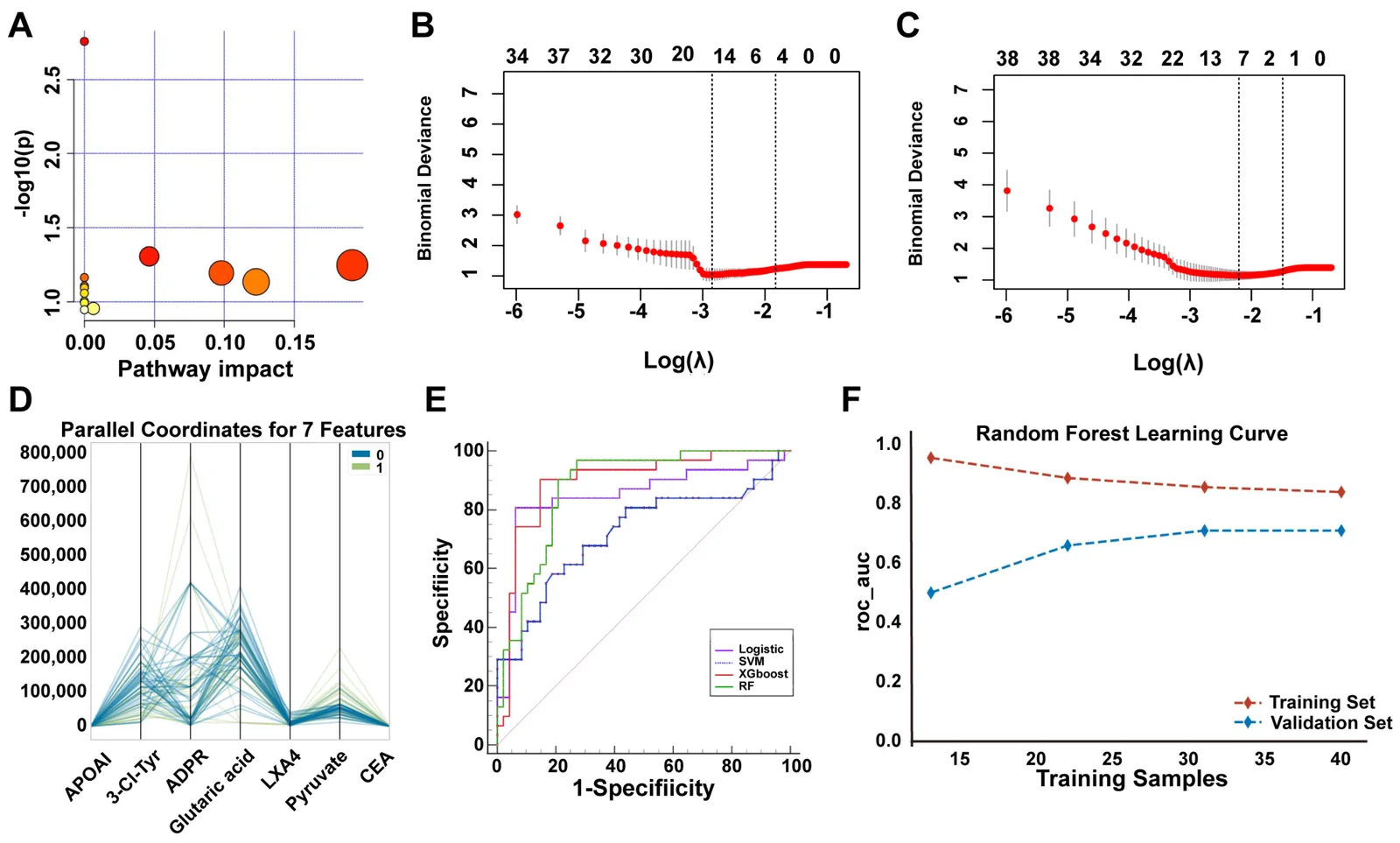
Variable selection and classifier comparisons. (**A**) Pathway enrichment; (**B**,**C**) LASSO penalty curves for clinical variables and bile metabolites; (**D**) the seven selected features; (**E**) classifier ROC curves; and (**F**) random-forest learning curve. In (**A**), circle size represents pathway impact and circle color represents *p* value (red: smaller *p*, yellow: larger *p*); in (**B**,**C**), vertical dashed lines indicate the selected lambda values.

The selected signature comprised pyruvic acid, lipoxin A4, CEA, APOA1, 3-chloro-L-tyrosine, ADP-ribose, and glutaric acid (Figure 5B–D). CA19-9 was considered as a candidate serum marker but was not retained; CEA was retained.

### 3.4. Random Forest Performance in the Study Cohort

**Figure 6:**
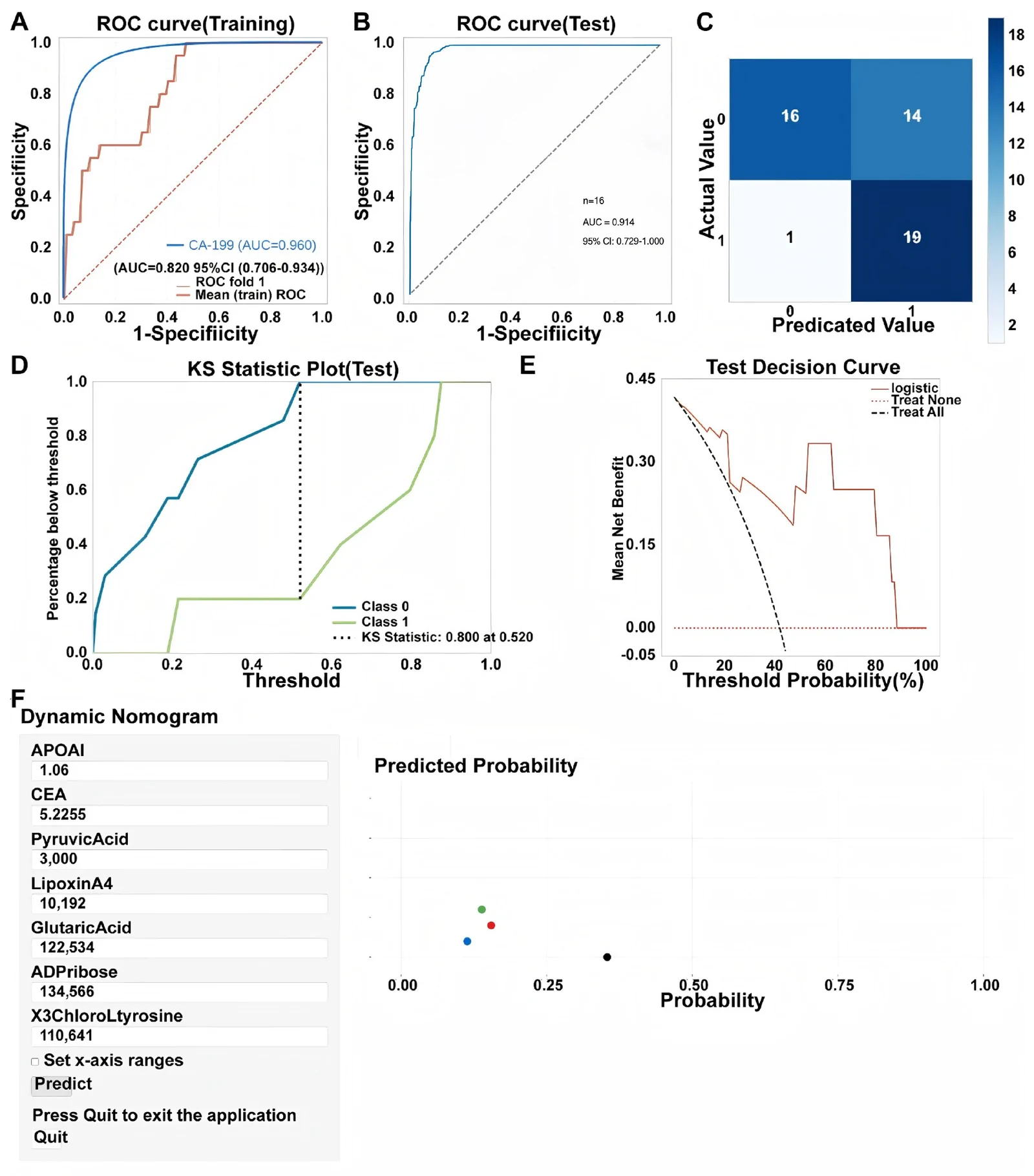
Internal model evaluation. (**A**) Training cross-validation ROC for the seven-variable panel (*n* = 63; AUC = 0.820, 95% CI 0.706–0.934), with CA19-9 from a separate 50-patient complete-case analysis (AUC = 0.960) overlaid for descriptive context, not a paired comparison. (**B**) Panel ROC in the 16-patient test subset (AUC = 0.914, 95% CI 0.729–1.000). (**C**) Confusion matrix for 50 complete cases, distinct from the test subset in (**B**). (**D**) KS plot; (**E**) decision-curve analysis; and (**F**) research-use online calculator. In (**A**,**B**), the dashed diagonal line represents the random-guessing reference (AUC = 0.5); in (**F**), colored dots indicate predicted probabilities from the online nomogram.

Four candidate classifiers were compared: random forest, support vector machine, XGBoost, and logistic regression. Random forest showed the highest AUC in the classifier comparison (0.894; Figure 5E). For the final seven-variable classifier, the reported training cross-validation AUC was 0.820 in 63 patients (Figure 6A), and the AUC in the 16-patient test subset was 0.914 (95% CI 0.729–1.000; Figure 6B). We present the training cross-validation result as the main internal estimate and the small test–subset result separately. As explained in Methods, feature selection preceded the split and may make these internal estimates optimistic.

Figure 6B shows the ROC for the 16-patient test subset. The confusion matrix in Figure 6C contains 50 complete cases (16 true negatives, 14 false positives, 1 false negative, and 19 true positives) and is reported separately from the 16-patient ROC. Figure 6D presents the KS plot, and Figure 6E presents the decision-curve analysis.

CA19-9 alone had a reported AUC of 0.960 in a 50-patient complete-case analysis and is overlaid in Figure 6A for context. Because the seven-variable panel AUC of 0.820 in that panel comes from training cross-validation, the two curves do not provide a paired statistical comparison. The test ROC in Figure 6B does not include a CA19-9 curve. Thus, these results do not establish added diagnostic value over the routine serum marker.

The seven-variable model and its research-use online calculator provide a basis for further evaluation of bile and serum markers in this clinical setting. The research-use calculator link is https://my-app123.shinyapps.io/dynnomapp/ (accessed on 7 October 2026) (Figure 6F).

### 3.5. SHAP Analysis of Model Feature Contributions

**Figure 7:**
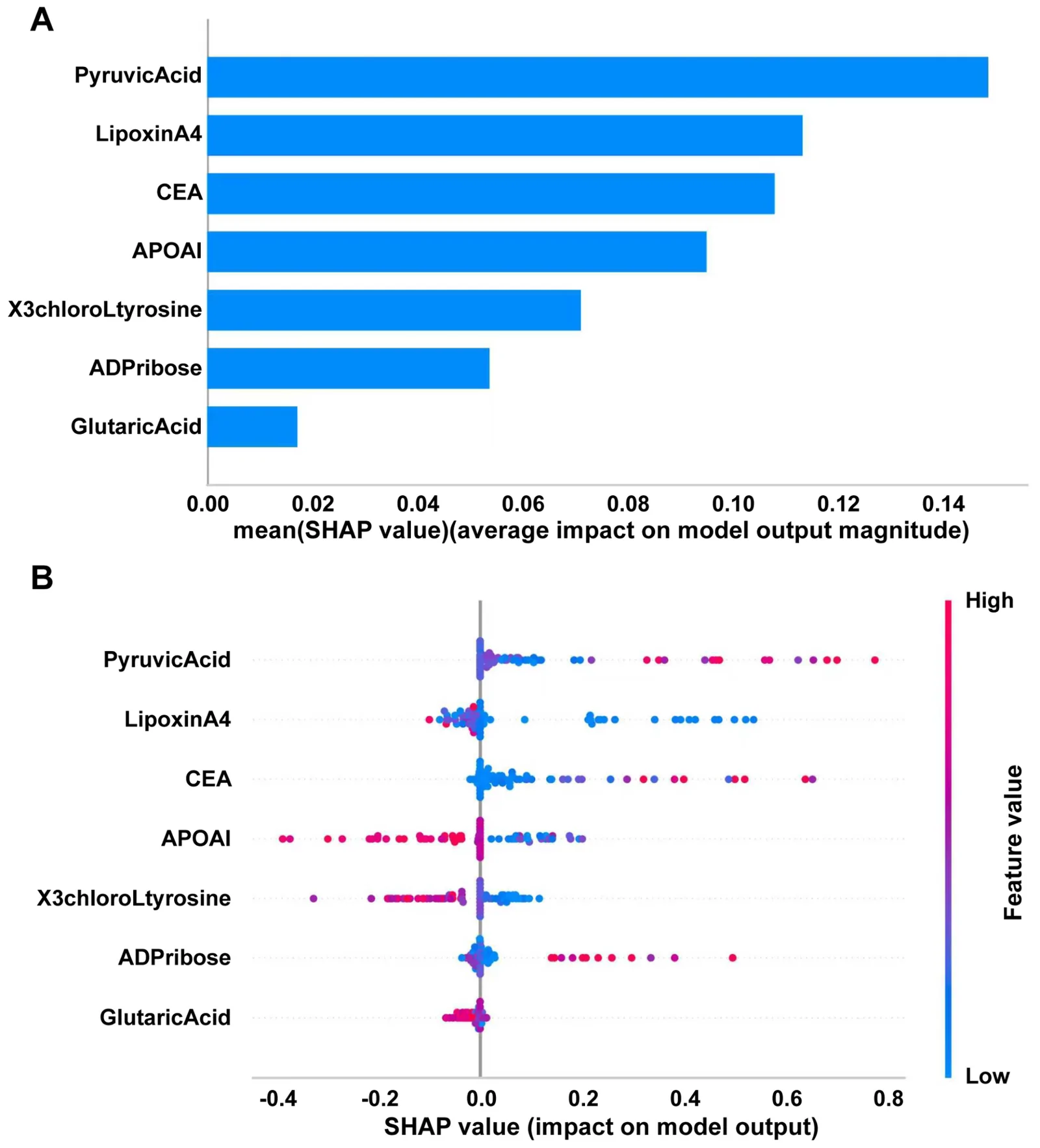
SHAP Analysis of the Seven-Variable Model. (**A**) Global feature contributions ranked by mean absolute SHAP value. (**B**) Summary plot showing the direction and magnitude of each variable’s contribution to random-forest predictions.

To examine model interpretability, we performed SHapley Additive exPlanations (SHAP) analysis on the final RF classifier. Global SHAP ranking showed that pyruvic acid made the largest contribution to model output, followed by lipoxin A4, CEA, APOA1, 3-chloro-L-tyrosine, ADP-ribose, and glutaric acid (Figure 7A). The SHAP summary plot further illustrated the direction and magnitude of each feature’s contribution across individual samples (Figure 7B). Higher pyruvic acid and CEA values shifted the model output toward CCA prediction, whereas higher APOA1 values shifted the model output away from CCA. The enzyme- and pathway-associated network provides further context for the selected metabolites (Supplementary Figure S2).

### 3.6. Single-Cell Transcriptomics Identifies Glycolysis- and Pyruvate-Associated Programs in CCA Tissue

The tabulated sample inventory contains 21 samples from 13 independent donors across GSE213452 (7 donors), GSE138709 (5 donors), and GSE181878 (1 donor): 13 tumor and 8 adjacent-tissue samples, with listed cell counts summing to 173,494 (Supplementary Table S4). Donors do not overlap across these datasets. Pooled-cell comparisons treat cells, rather than independent donors, as the analytic unit; they are shown only as descriptive tissue context.

**Figure 8:**
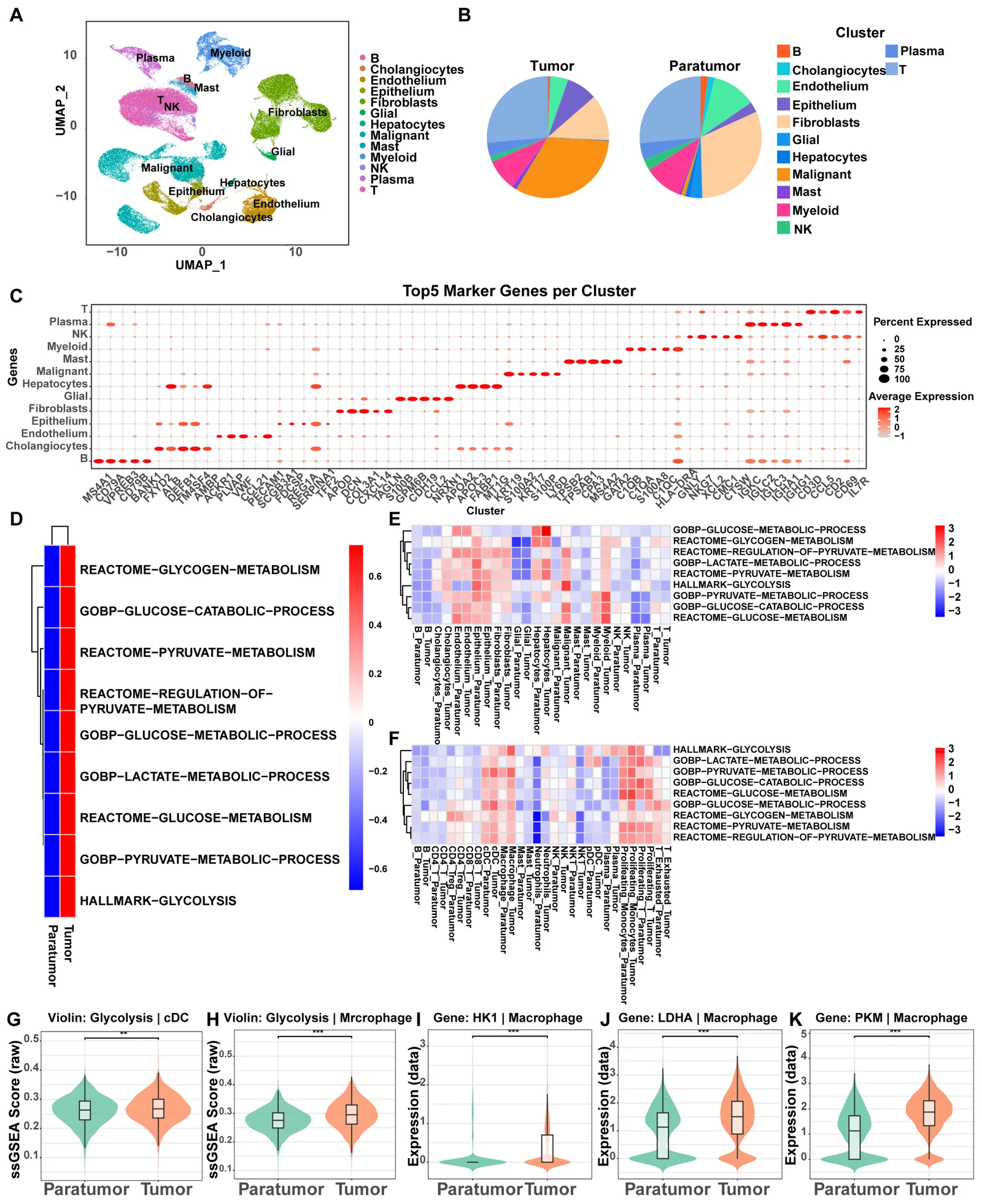
Single-Cell Transcriptomic Analysis of Glycolysis- and Pyruvate-Associated Programs in CCA. (**A**) UMAP of integrated single-cell data showing major cell populations in tumor and peritumor tissues. (**B**) Relative cell-type composition. (**C**) Representative cell-type marker genes. (**D**–**F**) Glycolysis- and pyruvate-related pathway scores across tissues and cell populations. (**G**,**H**) Glycolysis-related scores in dendritic cells and macrophages. (**I**–**K**) HK1, LDHA, and PKM expression in macrophages from tumor and peritumor tissues. ** *p* < 0.01, *** *p* < 0.001.

In the integrated analysis, glycolysis-related transcriptional patterns differed between tumor and peritumor cells (Figure 8D–K). Because these comparisons used pooled cells, the reported cell-level statistics are interpreted descriptively rather than as independent patient-level tests.

These single-cell findings provide supportive tissue context for glycolysis- and pyruvate-associated programs in CCA and are consistent with the model-prioritized pyruvate signal. They support biological plausibility, while the origin of bile pyruvate remains to be determined.

## 4. Discussion

In this retrospective cohort, bile metabolomics identified differences between CCA and cholelithiasis and supported a compact seven-variable model integrating bile metabolites with routine serum measurements. Random forest showed the strongest discrimination among the candidate classifiers in the internal comparison, and pyruvic acid was the leading contributor to model predictions. The tested comparison was CCA versus cholelithiasis; patients with indeterminate strictures, PSC, or IgG4-related cholangitis were not included as distinct control groups [29]. These findings provide a focused starting point for evaluating the signature in more challenging clinical comparisons.

Pyruvic acid was the leading model contributor, and the public single-cell data showed glycolysis- and pyruvate-associated transcriptional patterns in CCA tissue [30]. Together, these findings provide a biologically plausible context for the pyruvate component of the signature. The other selected bile metabolites and serum variables may capture complementary information across the local biliary environment; the present data do not assign each variable a single causal pathway [31,32,33,34,35].

The metabolomic findings also underscore why diagnostic translation requires more than identifying statistically different metabolites. Discovery-phase liquid-biopsy studies often generate long lists of candidate markers, but these signatures may lose performance when tested in larger and more heterogeneous cohorts because of statistical shrinkage, disease-stage variation, comparator heterogeneity, pre-analytical differences, analytical-platform effects, and biological context dependence [4,13,21,23]. This issue is particularly relevant for bile, where solute concentration, obstruction severity, inflammation, sample acquisition route, and procedural context may all influence measured metabolite abundance. Therefore, the translational value of the present analysis lies less in any individual differential metabolite than in the attempt to reduce a high-dimensional bile-metabolomic signal into a compact, clinically interpretable diagnostic framework. The seven-variable panel identified here, comprising pyruvic acid, lipoxin A4, CEA, APOA1, 3-chloro-L-tyrosine, ADP-ribose, and glutaric acid, is clinically plausible because it integrates local bile metabolites with routinely available clinical variables. This design also responds to a practical issue in liquid-biopsy development: broad multi-biofluid diagnostic systems may be difficult to implement at scale because of cost, specimen logistics, workflow burden, and platform harmonization requirements. The present model is therefore best understood as bile-centered rather than broadly multi-biofluid. Serum biochemical parameters and tumor markers were incorporated because they are already part of standard hepatobiliary evaluation, not because the model requires an additional experimental specimen stream. From a translational perspective, this distinction matters. A future clinically usable model would need to be analytically reproducible, compatible with quality-control procedures, sufficiently compact for implementation, and validated across centers and platforms. The present study does not yet establish such a deployable in vitro diagnostic test, but it provides a focused candidate framework for that next stage of validation.

Feature selection used all 79 patients before the training/test split, so the reported internal AUCs may be optimistic. Future analyses can repeat selection within resampling folds and assess the model in an independent cohort.

A future study could test whether bile profiling adds information in patients undergoing investigation of indeterminate strictures, including those with PSC or IgG4-related cholangitis [36]. Such patients were not included as controls here, so no diagnostic claim about them follows from this cohort.

The cohort was small and from one center, with cholelithiasis as the only benign diagnostic group; only two CCA cases were intrahepatic. Collection route, prior biliary intervention, and cholangitis differed between groups and may contribute to bile-metabolomic variation. One CCA case was classified clinically and by imaging without histologic confirmation. These factors, together with full-cohort feature selection and the absence of a same-patient serum-marker benchmark, limit conclusions about generalizability and added diagnostic value.

## 5. Conclusions

This study identified distinct bile-metabolomic patterns and a seven-variable bile and serum signature that differentiated CCA from cholelithiasis in the study cohort. The integrated model provides a promising candidate for further development. Because feature selection preceded internal evaluation, performance estimates require confirmation; added value over routine serum markers and performance against other benign biliary conditions remain to be tested.

## Figures and Tables

**Table 1:** Baseline characteristics of patients classified as CCA and of patients with cholelithiasis.

	CCA (*N* = 31)	Control (*N* = 48)	Statistic	*p*
Age (year), mean ± SD	66.4 ± 5.2	62.1 ± 2.0	t = 4.49	<0.001
Sex (male), *n* (%)	17 (54.8)	28 (58.3)	χ^2^ = 0.09	0.76
Hypertension, *n* (%)	8 (25.8)	17 (35.4)	χ^2^ = 0.80	0.37
Diabetes, *n* (%)	12 (38.7)	20 (41.7)	χ^2^ = 0.07	0.79
Total bilirubin (post-decompression, day of bile collection), µmol/L	14.2 ± 2.2	12.1 ± 3.5	t = 3.33	0.001
CCA site, *n*	2 intra/29 extra	—	—	—

## Data Availability

The datasets generated and/or analyzed during the current study are available from the corresponding author on reasonable request. Public single-cell RNA-sequencing datasets analyzed in this study are available in the Gene Expression Omnibus under accession numbers GSE213452, GSE181878, and GSE138709.

## Supplementary Materials

The following supporting information can be downloaded at https://www.mdpi.com/article/10.3390/biomedicines14102356/s1, Figures S1–S3 present analytical and single-cell quality-control and pathway-context plots. Supplementary Table S1 lists differentially expressed metabolites (VIP > 1, *p* < 0.05) between groups; Supplementary Table S2 lists all annotated metabolite features; Supplementary Table S3 reports CCA clinicopathologic features, and Supplementary Table S4 lists public single-cell samples.
